# Amputation of the Thigh, in a Case of Fungus Hæmatodes

**Published:** 1838-03-14

**Authors:** J. Randolph

**Affiliations:** Surgeon to the Hospital


### CLINICAL REPORTS.

### PENNSYLVANIA HOSPITAL.

*Amputation of the Thigh, in a case of Fungus Hæmatodes, by* J. Randolph, M. D., *Surgeon to the Hospital.*

A mulatto woman, twenty four years of age, was admitted into the Pennsylvania Hospital, 23d October, 1837, for a tumour occupying the whole of the lower part of the right thigh. She stated, that when twelve years of age, she felt a weakness in the right knee, and that, after much exertion, it became painful, but not to such a degree as to interfere with her ordinary household occupations. Eighteen months previous to her admission into the hospital, while carrying one of her children, she fell and hurt the affected limb; since which time, it gave her constant and increasing pain, and the swelling rapidly advanced. In July last, she suffered so much, as to be confined to her room, for several days; cups were, at this time, freely applied to the tumour, warm fomentations constantly used, some purgative medicines administered, and a bandage afterwards worn for some time. From all these remedies, she derived no benefit, and her symptoms were gradually aggravated. For the first time, she was now obliged to have recourse to a crutch, and, even with the aid of it, could with difficulty move about the house. On the 15th October last, while going up stairs, she fell upon the leg, and "felt it give way;" from which time, she was unable to stand upon it, and the least motion gave her excruciating pain. On the following week, she entered the hospital, at which time, her condition was as follows. The thigh, from the knee-joint, to within three inches of the groin,—a space of nine inches,—was occupied by a tumour, of an ovoid shape, the anterior semi-circumference of which was inferior in size to the posterior. The dimensions of the diseased limb, as compared with the healthy, were the following:

| | Inches in circumference. |
|---|---|
| One inch above the knee, | 19 |
| At the middle of the thigh, | 24 |
| Three inches from the groin, | 17 |

The healthy limb measured at these points, 12, 16, and 17 inches, respectively.

The tumour, when handled, offered an obscure feeling of fluctuation, and an indistinct crepitus could be perceived. There was no alteration in the colour of the skin, which was very hot, and did not appear to have contracted unnatural adhesions with the tumour. Cold lead water was ordered to be kept constantly to it; a purgative, every other day; and an opiate, at night, at which period, there was a slight paroxysm of hectic fever. Up to the 15th October, her general health had been perfect. It may be remarked, that she complained of more pain in the ankle, which was perfectly healthy, than in the tumour, in consequence, probably, of pressure on the nerves supplying that part.

*Wednesday*, 15*th November*. she was introduced into the amphitheatre, present Drs. Randolph, Harris, Norris, and Barton. Dr. Randolph gave it as his opinion, that the disease was probably Fungus Hæmatodes; but, as the inguinal glands did not appear to be at all affected, and the woman enjoyed a good constitution, which was unimpaired, except during the short time she had laboured under hectic fevor, a removal of the limb was justifiable, as it afforded the only chance of prolonging life. The patient's sufferings were so great, that she was very anxious to have something done for her relief.

Previous to the operation, in order to ascertain the contents of the tumour, an opening was made into it with a lancet, from which a large discharge of blood took place. The wound was closed with lint, after which, Dr. Randolph proceeded to perform the amputation. A circular incision was made, and the skin to form the flap appearing perfectly healthy, it was dissected from the tumour, below. The bone was sawed three inches from the groin. The muscles, when cut, were of a paler colour than natural. Eight ligatures were applied.

Dr. R. stated to the class, that, from the appearance of the stump, he had little hope of a permanent cure taking place, as it was probable the disease had progressed so far, as to involve the neighbouring tissues. The tumour, when opened, was found filled with cells, containing a greenish fluid, with but little odour. The solid parts of the tumour were composed of a whitish medullary substance, with numerous spicula of bone in it. It appeared to have grown from the bone, and was six inches in length, and five in width. The femur itself had the appearance of having been grated away, for a distance of an inch in length, and had the sides of the two extremities rubbed off for two inches. This accounts for the crepitus, which was heard, at the woman's admission, and it is probable that a fracture occurred, upon the receipt of the last injury, above mentioned.

The patient was discharged on the 10th of January, the stump having healed, except for the space of a quarter of an inch, and the woman being anxious to be at home. I visited her four days after, and found the stump perfectly firm. She has since menstruated with regularity, suffers no pain, and enjoys excellent health. Even should a return of the disease take place, the advantage of an operation is most satisfactorily shown, as the woman would otherwise have been, in all probability, worn out before this, by the disease.

*March* 8*th*. Visited the patient to-day, more than sixteen weeks since the operation. There is no appearance whatever of a return of the disease, and the woman enjoys perfect health. Any future change in her condition will be reported.

J. M. Wallace, M. D. Resident Surgeon.

